# Supplementary material for: DanioCTC: Analysis of Circulating Tumor Cells from Metastatic Breast Cancer Patients in Zebrafish Xenografts
Source: Cancers (Basel). 2023 Nov 14;15(22):5411. doi: 10.3390/cancers15225411 (PMC10670801; doi:10.3390/cancers15225411)
Supplement: Supplementary file 1 [file cancers-15-05411-s001.zip › cancers-2548871-supplementary.pdf]

Supplement

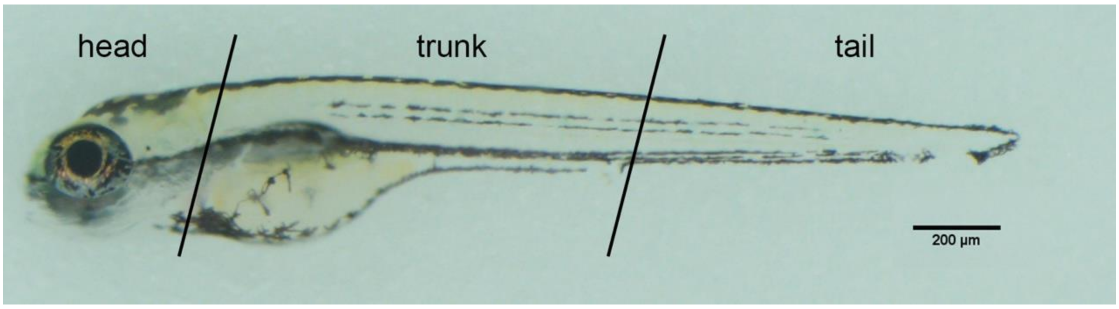

Supplement Figure 1: Regions of the zebrafish larva: The larval body was divided into three regions: head, trunk, and tail for cell count analysis.

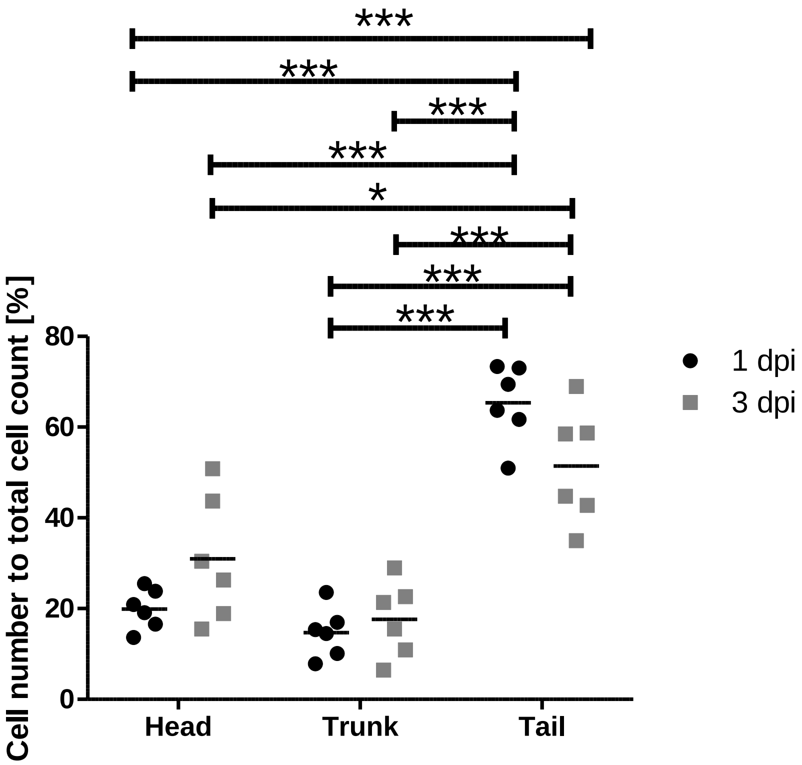

Supplement Figure 2: Dissemination of MDA-MB-231 cells after injection with the standard workflow into 2 dpf zebrafish larvae. Depicted are the relative numbers of MDA-MB-231 cells localized in the head, trunk and tail regions at 1 and 3 dpi (n=6). 19.9 % of the cells were located in the head, 14.7 % of cells in the trunk and 65.3 % in the tail at 1 dpi. At 3 dpi, 30.9 % of the cells were located in the head region, 17.6 % in

the trunk and 51.4 % in the tail. dpi: days post injection, ANOVA followed by post-hoc Bonferroni test, *0.01 < p < 0.05; ***0.0001 < p < 0.001

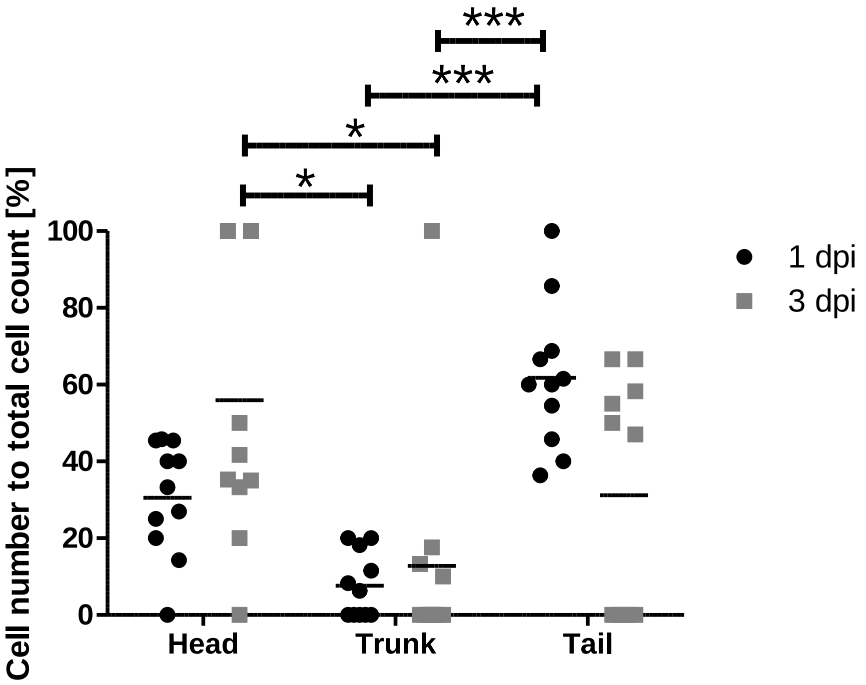

Supplement Figure 3: Dissemination of MDA-MB-231 cells spiked into DLA samples after injection with DanioCTC workflow into zebrafish larvae. Depicted are the relative numbers of MDA-MB-231 cells localized in the head, trunk and tail regions at 1 and 3 dpi (n=11). 30.5 % of the cells were located in the head, 7.6 % of cells in the trunk and 61.7 % in the tail at 1 dpi. At 3 dpi, 55.9 % of injected cells were detected in the head, 12.8 % in the trunk and 31.2 % in the tail. dpi: days post injection, Kruskal-Wallis followed by post-hoc Dunn's test, *0.01 < p < 0.05; ***0.0001 < p < 0.001

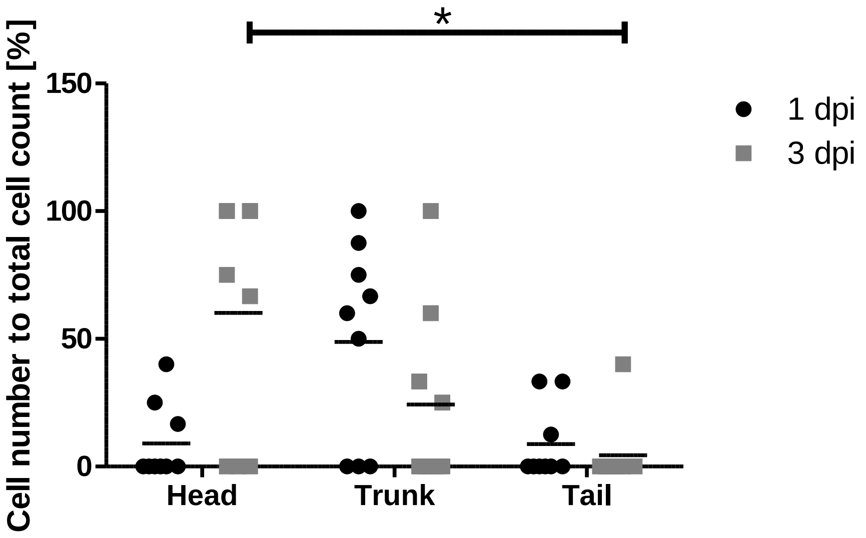

Supplement Figure 4. Dissemination of isolated CTCs of a MBC patient after injection with DanioCTC workflow into zebrafish larvae. Depicted is the relative CTC dissemination into the head, trunk and tail at 1 and 3 dpi (n=9). 9% of the CTCs were present in the head, 48,7 % in the trunk and 8,7% in the tail at 1 dpi. At 3 dpi, their numbers corresponded to 60.1%, 24.2%, 4.4% in the head, trunk and tail, respectively. dpi: days post injection. Kruskal-Wallis test followed by post-hoc Dunn's test, *$0.01 < p < 0.05$
